# The secretome of *Agaricus bisporus*: Temporal dynamics of plant polysaccharides and lignin degradation

**DOI:** 10.1016/j.isci.2023.107087

**Published:** 2023-06-09

**Authors:** Katharina Duran, Joris Magnin, Antoine H.P. America, Mao Peng, Roelant Hilgers, Ronald P. de Vries, Johan J.P. Baars, Willem J.H. van Berkel, Thomas W. Kuyper, Mirjam A. Kabel

**Affiliations:** 1 Laboratory of Food Chemistry, Wageningen University & Research, Bornse Weilanden 9, 6708 WG Wageningen, the Netherlands; 2 Bioscience, Wageningen University & Research, Droevendaalsesteeg 1, 6708 PB Wageningen, the Netherlands; 3 Fungal Physiology, Westerdijk Fungal Biodiversity Institute & Fungal Molecular Physiology, Utrecht University, Uppsalalaan 8, 3584 CT Utrecht, the Netherlands; 4 CNC Grondstoffen, Driekronenstraat 6, 6596 MA Milsbeek, the Netherlands; 5 Soil Biology Group, Wageningen University & Research, Droevendaalsesteeg 3a, 6708 PB Wageningen, the Netherlands

**Keywords:** Microbiology, Biotechnology, Biomass

## Abstract

Despite substantial lignocellulose conversion during mycelial growth, previous transcriptome and proteome studies have not yet revealed how secretomes from the edible mushroom *Agaricus bisporus* develop and whether they modify lignin models *in vitro*. To clarify these aspects, *A. bisporus* secretomes collected throughout a 15-day industrial substrate production and from axenic lab-cultures were subjected to proteomics, and tested on polysaccharides and lignin models. Secretomes (day 6–15) comprised *A. bisporus* endo-acting and substituent-removing glycoside hydrolases, whereas β-xylosidase and glucosidase activities gradually decreased. Laccases appeared from day 6 onwards. From day 10 onwards, many oxidoreductases were found, with numerous multicopper oxidases (MCO), aryl alcohol oxidases (AAO), glyoxal oxidases (GLOX), a manganese peroxidase (MnP), and unspecific peroxygenases (UPO). Secretomes modified dimeric lignin models, thereby catalyzing syringylglycerol-β-guaiacyl ether (SBG) cleavage, guaiacylglycerol-β-guaiacyl ether (GBG) polymerization, and non-phenolic veratrylglycerol-β-guaiacyl ether (VBG) oxidation. We explored *A. bisporus* secretomes and insights obtained can help to better understand biomass valorization.

## Highlights

• Secretomes of *A. bisporus* comprise a diverse set of lignin active enzymes
• Modifications of dimeric lignin models were catalyzed by *A. bisporus* secretomes
• Prevalence of UPOs might play a role in *A. bisporus*-mediated delignification
• Protein composition of axenic and industrial scale *A. bisporus* secretomes match

## Graphical abstract

**Figure:**
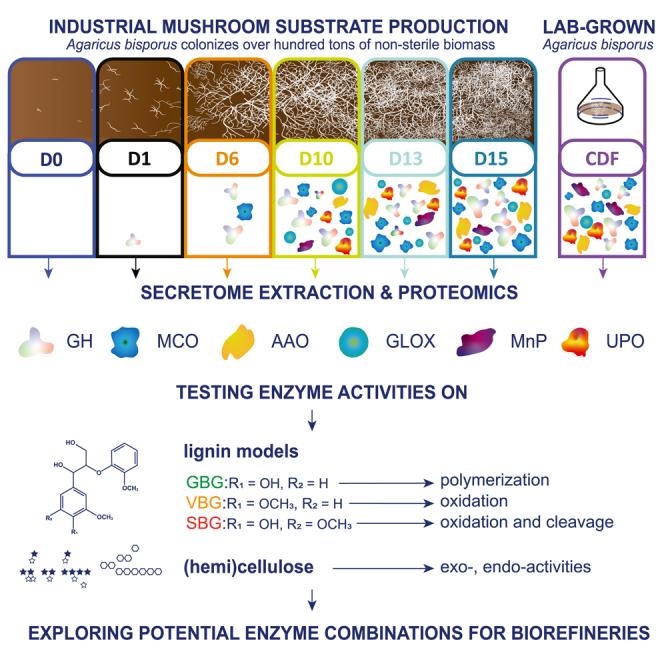

## Introduction

In nature, plant biomass degradation is ubiquitous.^1^ Especially in biodiverse, natural systems inhabited by fungi and bacteria, the turnover of plant biomass can be high and even recalcitrant lignin-rich biomass is targeted, degraded, and catabolized.^2^^,^^3^^,^^4^^,^^5^^,^^6^ Endeavors to moving to a bio-based economy require efficient valorization of lignocellulosic agricultural (by)-products and commercial-scale valorization.^7^^,^^8^ Biotechnological approaches can be excelled by harnessing species diversity that is present in natural systems.^9^ The complexity of such systems, however, makes the search for specific lignocellulose degrading enzymes difficult. A simpler approach is to explore the functional diversity in existing fungal-driven lignocellulose conversion environments, such as in the commercial substrate production process for the white button mushroom, *A. bisporus*.^10^ This process is interesting as it is effective in converting low-value lignocellulose (composed of wheat straw, horse and chicken manure and gypsum), to a food product.^11^

The white button mushroom production process is composed of four distinct phases (PI, PII, PIII, PIV) and PI – PIII take place in non-sterile conditions in tunnels containing over hundred tons of lignocellulosic material.^10^^,^^12^ Although the microbial interplay is considerably important throughout this process,^13^^,^^14^ *A. bisporus* dominates during the mycelial growth phase (PIII) and fruiting body formation phase (PIV).^15^^,^^16^ During these *A. bisporus* dominated phases, (hemi-)cellulose is broken down to smaller chains and removed by 20%,^12^ whereas approximately 40% of the lignin is removed from the substrate.^12^^,^^16^^,^^17^ Hence, we hypothesize that *A. bisporus* secretomes derived from this ligninolytic environment will reveal new insights in the (synergistic) action of enzymes, particularly targeting lignin, involved in this industrial bioconversion process.

*A. bisporus* is well-known to encode, translate, and produce a plethora of carbohydrate active enzymes (CAZymes; cazy.org),^18^ as well as oxidoreductases, relevant for its adaptation to the humic leaf-litter environment.^19^^,^^20^^,^^21^ Still, *A. bisporus* is not considered a typical lignin degrader, such as white-rot fungi.^6^^,^^22^^,^^23^ While white-rot fungi encode and produce a multitude of peroxidases, generally considered essential for delignification,^24^^,^^25^^,^^26^ the *A. bisporus* genome solely contains two manganese peroxidases (MnP).^27^ Nevertheless, the substantial delignification observed throughout *A. bisporus* mycelial growth^12^^,^^16^ indicates that other oxidoreductases may have a role in delignification, especially in environments where partly delignified compost-pretreated biomass is prevalent.^21^^,^^28^

Previous research provided indications that multicopper oxidases, and particularly laccases, are associated to be responsible for the lignin degradation by *A. bisporus*.^27^^,^^29^ However, redox potentials of fungal laccases are likely too low to directly oxidize non-phenolic lignin,^30^^,^^31^ and substantial delignification can only occur via so-called laccase mediator systems.^32^ Other leaf-litter degraders that are equipped with laccases have also successfully delignified litter,^33^ but currently leaving the question about these natural mediators unanswered.^34^ Besides the upregulation of laccases during *A. bisporus* mycelial growth, Morin et al*.* (2012) have pointed out that *A. bisporus* encodes a set of fairly uncharacterized heme-thiolate peroxidases – aromatic peroxygenases (HTP-APO) or, in short, unspecific peroxygenases (UPOs).^21^^,^^35^^,^^36^ So far, it has been proposed that UPOs have a versatile substrate scope, and some cleave ether linkages.^37^ A study from Kinne (2011) even showed cleavage of a dimeric non-phenolic lignin model by an UPO from *Agrocybe aegerita*.^38^ Owing to their versatile functions and potential involvement in ligninolysis, and as they have been reported to be solely produced at the end of PIII,^19^^,^^21^^,^^39^ we here investigated their earliest secretion and temporal changes during *A. bisporus* mycelial growth. Accessory oxidoreductases encoded in the *A. bisporus* genome,^21^ such as aryl alcohol oxidases (AAOs),^40^ glyoxal oxidases (GLOXs),^41^ and even laccases under certain conditions,^42^ might produce the required hydrogen peroxide as co-substrate for MnPs and UPOs.

We investigated (1) the relationship between oxidoreductases and delignification and (2) whether lignocellulose-degrading CAZymes, are not only encoded in *A. bisporus* genome, but also actively secreted. Furthermore, we focused (3) on temporal dynamics of the secretomes throughout *A. bisporus* mycelial growth in an industrial substrate production process and compared these to axenic lab-cultivated *A. bisporus* secretomes. To support proteomics, the secretomes (4) were tested for their ability to degrade plant polysaccharides and particularly modify dimeric lignin model compounds.

## Results and discussion

### Secretome compositions from industrial mycelial and axenic *A. bisporus*

**Figure 1:**
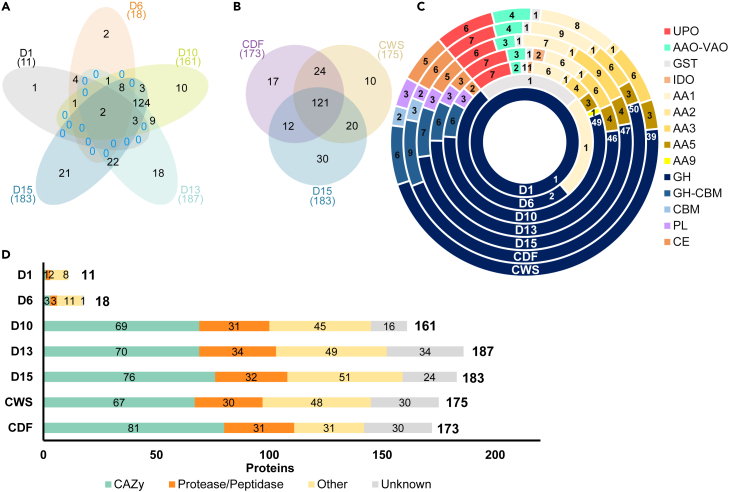
Functional distribution of *A. bisporus* secretomes (A) Venn diagram of all proteins (based on protein ID) found in the secretomes of *A. bisporus* colonized PIII substrate of day 1, 6, 10, 13 and 15 (D1, D6, D10, D13, D15). (B) Venn diagram of all proteins found in the secretomes of axenically cultivated A15 on PIII-start substrate (CWS), and on dark film medium (CDF), compared with that of (non-axenic) D15. (C) Number of proteins in the secretomes of the major found polysaccharide degrading CAZy-families and oxidoreductase-groups. Abbreviations stand for: UPO = unspecific peroxygenase; AAO-VAO = aryl alcohol oxidase, vanillyl-alcohol oxidase; GST = glutathione S-transferase; IDO = intradiol dioxygenase; AA 1–3, 5, 9 = auxiliary activities 1-3, 5, 9; GH = glycoside hydrolase; CBM = carbohydrate-binding module, PL = polysaccharide lyase, CE = carbohydrate esterase. (D) Number of proteins found in all secretomes categorized as CAZymes, Proteases/peptidases, Other and Unknown.

Secretomes were collected throughout the mycelial growth phase of the commercial substrate production of *A. bisporus* at day 1, 6, 10, 13 and 15. These sampling time points were chosen based on industrial experience of this process, that *A. bisporus* mycelium starts to become visible on day 6 in the substrate and from day 10 onwards substantial growth of the fungus is normally observed (i.e., based on visual appearance). Overall substrate changes related to cellulose, hemicellulose and lignin were in line with previous research assessing beginning and end material of similar industrial *A. bisporus* growth settings^12^^,^^16^^,^^17^*,* as cellulose, hemicellulose and lignin decreased throughout mycelial growth, with 20%, 18% and 40% (w/w), respectively (Table S2). The protein content per moist substrate material first decreased (2 μg/mg in D0, 1.76 μg/mg in D1, 1.49 μg/mg, in D6, 1.03 μg/mg in D10) and then increased (1.6 μg/mg in D13, 3.49 μg/mg in D15). However, the functional distribution of proteins (Figure 1A) clearly indicated a gradual increase of the number of *A. bisporus* proteins found in the secretomes D1 up to D15, even with equal protein concentration kept for proteomic analysis.

In the beginning of *A. bisporus* mycelial growth phase, only 11 (D1) and 18 (D6) *A. bisporus* proteins were found in the secretomes, whereas an almost 10-fold increase occurred in the second half of the mycelial growth phase. In the D10 secretome already 161 proteins were identified, increasing to 187 (D13) and to 183 (D15). A similar number of proteins were identified in axenically grown *A. bisporus* secretomes (175 and 173), of which one was cultivated on PIII-start material (CWS) and the other one on dark-film medium (CDF) (Figure 1B). This gradual increasing number of *A. bisporus* proteins was expected, as the fungus was introduced in the substrate at the beginning of PIII. We found 99 proteins with SignalP values higher than 0.75, which are therefore assumed to be secreted and not because of e.g., cell lysis (Table S1).

D10, D13 and D15 showed a considerable overlap of 124 proteins, and a similar overlap of 121 proteins between D15 and the axenically grown CWS and CDF was observed (Figure 1B). These high overlapping numbers between the compost and axenic samples confirms that the detected proteins in the non-sterile industrial-scale substrate secretomes correspond to proteins produced by *A. bisporus*, and other microbial proteins in PIII-secretomes did not interfere with our proteomics analysis.

In the secretomes, we could distinguish four main groups: Proteases/peptidases, CAZymes, Other (e.g., various oxidoreductases) and Unknown proteins. Around 30 *A. bisporus* proteins were assigned to the group of Proteases/peptidases in the later secretomes (>D10; Figure 1), as well as in CWS and CDF. Hence, Proteases/peptidases made up 15%–19% of the secretomes. The high number of Proteases/peptidases found may be related to the environment in which *A. bisporus* thrives, which is a (pre-composted) microbial biomass and humic substance-rich environment in this study. Proteases are essential to assure nitrogen recycling, for example retrieving it from microbial necromass and from humic substances.^43^ Contrarily, in secretomes obtained from axenically cultivated white-rot fungi grown on non-composted lignocellulose substrates, only 7% (*Pleurotus eryngii*)^44^ or 2% (*Podospora anserina*)^45^ of all secretomic proteins were found to be proteases/peptidases.

The carbohydrate active enzymes (CAZymes) largely dominated the functional diversity of the proteins (Figure 1D). From day 10 onwards around 40% of the proteins were identified as CAZymes in the secretomes (D10-15). The secretomes contained up to 19% of the total number of 423 CAZymes encoded in the *A. bisporus* H97 genome. Secretomes from later stages (D10-D15) had similar numbers of CAZymes in comparison to the axenic secretomes where 80 (CDF) and 67 (CWS) proteins were identified as CAZymes. The glycoside hydrolase (GH) class was the most abundant CAZyme class with 49, 46 and 47 proteins in D10, D13 and D15, respectively (Figure 1C). In addition, CAZy members categorized as auxiliary activities (AAs), were abundant in the secretomes. The most represented AA family was AA1, comprising multicopper oxidases (MCOs) and some *sensu stricto* laccases. Furthermore, four glyoxal oxidases (AA5_1), four aryl alcohol oxidases (AA3_2), one LPMO (AA9), and one manganese peroxidase (AA2) were identified in the *A. bisporus* secretomes. Like for the GHs, the number of AAs increased in the later secretomes. In the D6 secretome only one AA protein was identified, which increased to 15 in D10, 17 in D13 and 21 in D15. Again, similar numbers were found in the axenic secretomes, with 19 AA proteins in CDF and CWS each. In total, the *A. bisporus* genome encodes 82 AAs and in the secretomes 27 different AAs were identified.^21^

Around 30% of the proteins in the *A. bisporus* secretomes have been assigned to Other putative functions (according to Pfam), including lipases, FAD-binding proteins, dioxygenases, choline esterases, etc. Within proteins categorized as Other, we also found putative UPOs in the secretomes from the industrial-scale substrate production (6–7 in >D10; Figure 1C) and in axenically cultivated *A. bisporus* secretomes (6 in CWS and 7 in CDF; Figure 1C). They belong to an interesting group of oxidoreductases that have been associated with ligninolysis.^21^^,^^35^^,^^39^ Still around 10 and 20% of the proteins in the secretomes have no assigned function, which is within a normal range (6–30%).^44^^,^^45^

We can clearly see an increase in intensity based absolute quantification (iBAQ) of enzymes acting on lignin, aromatics, and on proteins, and to a lesser extent of enzymes acting on cellulose and hemicellulose (Figure S1). These findings are in line with previously observed substrate changes,^12^ and substrate changes observed in this experiment (Table S2) where cellulose and hemicellulose were degraded, but removed to a much lesser extent than lignin.

### Temporal dynamics of secretomes related to polysaccharide degradation

**Figure 2:**
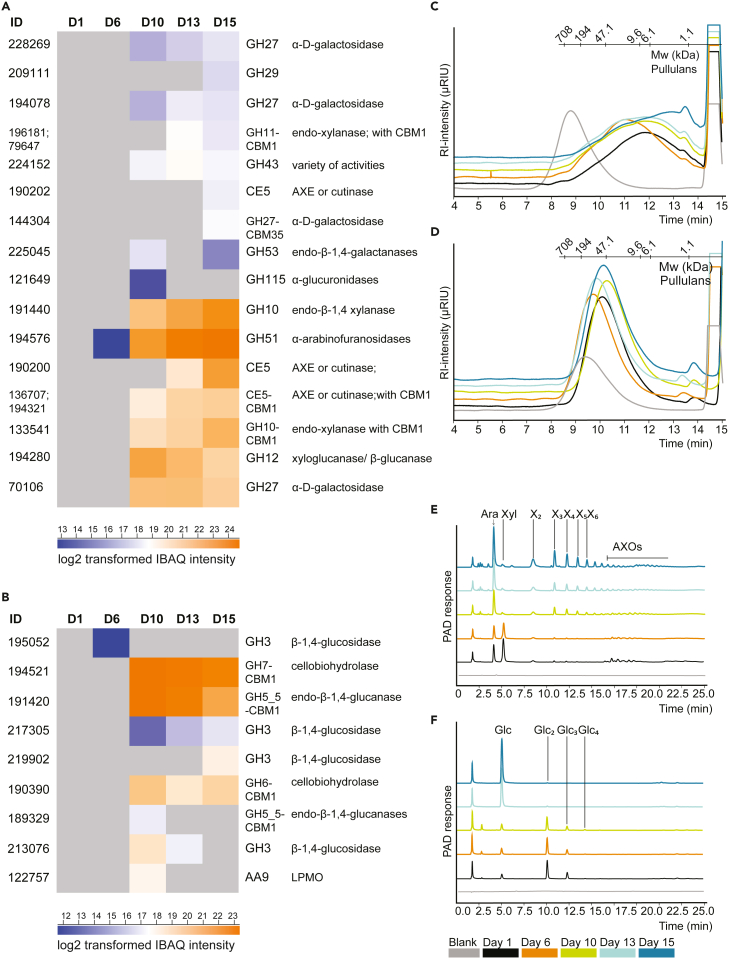
Secretomic profiling of hemicellulosic and cellulosic digests (A–F) Protein IDs and log2 transformed IBAQ intensities in D6, D10, D13 and D15 related to hemicellulose, xylan (A), and cellulose (B) degradation. Results for other CAZymes are presented in Figure S4. CAZymes family and subfamily, protein ID and putative functions are provided if available and based on Joint Genome Institute identifier ‘jgi|Agabi_varbisH97_2’and Billeti et al.^21^ GH = glycoside hydrolase; CBM = carbohydrate-binding module; AA = auxiliary activities; CE = carbohydrate esterase. Secretomes were used to digest carboxymethyl cellulose (CMC) and wheat arabinoxylan (WAX) and corresponding product profiles were analyzed by high performance size exclusion chromatography (C=WAX, D = CMC) and high-performance anion exchange chromatography (E = WAX, F=CMC). Ara = arabinose, Glc = Glucose, Xyl = xylose, X_2_ – X_6_ = xylose dimer – hexamer, AXOs = xylo-oligomers substituted with arabinose.

In all secretomes, the GHs that were identified were mainly related to xylan, cellulose, and chitin degradation (Figures 2; S2 and S3). Therefore, the secretomes were tested for their catalytic ability toward wheat arabinoxylan (WAX; Figures 2C and 2E), birchwood glucuronoxylan (now referred to as xylan), xyloglucan, chitin, and galactomannan (Figures S2 and S3).

Arabinoxylan degradation started between day 6 and day 10 and in total decreased by 18% (w/w) (Table S2). In relation to arabinoxylan degradation, the most abundant protein in D15 was a hypothetical α-arabinofuranosidase (GH51; ID 194576). This protein was already present in D6 and increased gradually in the secretomes of the later stages. The same protein was found in the secretome obtained at the end of PIII by Patyshakuliyeva et al. (2015).^19^ Indeed, arabinose release from WAX with the various secretomes was observed, and this arabinose release was higher for digests of WAX with D10, D13 and D15 compared with those with D1 and D6 (Figure 2) when assayed at the same pH and temperature. An opposite trend was observed for xylose, which was released from WAX and xylan more extensively with D1 than with D10, D13 and D15 secretomes (Figures 2 and S3), at least when assayed at the same pH (pH 5.5) and temperature (35°C). Acidification during mycelial growth (at 25°C^10^) was observed (from pH 7.4 to 6.2), and to stay close to these conditions, we decided to assay the enzymes at a slightly acidic (but buffered) pH of 5.5, and slightly elevated temperature (35°C). These results were in line with the 4-nitrophenyl (*p*NP)-β-d-xylopyranoside assay, which showed a maximum of over 1000 nmol *p*NP generation by D1, gradually declining toward a minimum of 22 nmol *p*NP by D10 (Figure S7). Solely one *A. bisporus* β-xylosidase protein (ID 219902) in D15 and CDF was picked up. Nonetheless, a few putative GH31 proteins with potential xylosidase activity (IDs: 183688, 190944, 75421 and 64273) (Figure S4) were detected as well in CDF and D15. Possibly, the xylosidase activity of early stage secretomes (D1) was not only related to *A. bisporus* enzymes, but other microbial enzymes (i.e., present in the substrate at the start^28^) which might have contributed to this activity as well.

The second main proteins picked up in relation to hemicellulose (xylan) degradation were putative endo-β-1,4 xylanases (GH10, ID 191440; GH10-CBM1, ID 133541; GH10, ID 191440 and GH11-CBM1, ID 196181&79647), in particular in D10, D13 and D15. Indeed, corresponding digests with WAX and xylan showed degradation of the polymer and formation of xylo-oligomers (Figures 2 and S3). In a previous study by Jurak (2015),^46^ endo-xylanases were also found in secretomes obtained from the end of PII and from PIV. Although endo-xylanase activity was observed throughout the whole mycelial growth phase of *A. bisporus,* based on HPSEC (Figure 2C), *A. bisporus* endo-active enzymes (Figure 2A) were only found from D10 onwards. It is plausible that an enzymatic replacement occurred, with endo-xylanase produced by *A. bisporus* from day 10 onwards replacing endo-xylanases produced by microbes that were dominant in PII and that remained present at the beginning of PIII (Figure 2A).

In addition to xylan-degrading enzymes, also galactomannan and xyloglucan GHs were identified, both as proteins in the secretomes (β-mannosidases - GH5_9, IDs 196962, 189449; xyloglucanases - GH16, IDs 194618, 195916, 194297 and β-1,4 galactosidase GH35 – ID 152299), and based on their endo-activities digesting these polysaccharides (Figures S2 and S3).

Overall, our findings hint at the presence of minor amounts of exo-acting hemicellulases, whereas endo-acting GHs were more pronounced, at least at the conditions used mimicking the conditions in the commercial substrate process (PIII). These findings largely matched with previous data showing minor saccharification of hemicellulose from the substrate in corresponding PIII end samples (D15 compared to D1). Still, D15 substrate previously showed a decrease in molecular weight of extracted xylan populations, which matched very well with the here observed endo-xylanase activities.^19^^,^^46^

Cellulose degradation reached an extent of 20% (w/w) and was highest between day 10 and day 13 (Table S2). The two most abundant cellulolytic proteins in D6 up to D15 were a putative cellobiohydrolase (CBH; GH7-CBM1, ID 194521) and a putative endo-glucanase (GH5-CBM1, ID 191420; Figure 2), which confirms results from Patyshakuliyeva.^19^ Furthermore, two other putatively annotated CBHs (GH6-CBM1, ID 190390 and GH7-CBM1, ID 194521) and a putative endo-glucanase (GH5_5-CBM1, ID 189329) were present in D10 up to D15. Similarly, several putative β-1,4-glucosidases from GH3 (IDs: 195052, 217305, 213076) were detected, whereas one GH3 (ID 195052) was present already in D6 (Figure 2). Although the MW decrease of carboxymethyl-cellulose (CMC) was minor, HPSEC results were still hinting at endo-glucanase activity under the conditions assayed, which correlates with the proteomics data, as one endo-glucanase was present in D10, D13 and D15 (GH5-CBM1, ID 191420; Figure 2). Release of glucose, cellobiose, cellotriose and cellotetraose from CMC was detected by HPAEC (Figure 2F). The release of cellobiose was highest in incubations with D6 and remained high until the end of PIII, corroborating with cellobiohydrolase activity (Figures 2A and 2F). Temporal dynamic changes of the secretomes were evident, as measurements at D0 and D6 showed the release of glucosyl units, whereas measurements at D10 onwards showed release to a minor extent. Similarly, β-glucosidase exo-activities were demonstrated on the chromogenic substrate 4-nitrophenyl-β-d-glucopyranoside, which was high in the beginning of PIII and from day 10 onwards remained low until the end of PIII (Figure S7). However, observed activities on CMC and *p*NP-substrates by D0, D1 and D6 were likely the result of enzymes that were produced by other microorganisms present in non-sterile industrial scale environment,^47^ as our proteomics data indicated the absence of *A. bisporus* cellulolytic enzymes until D10.

Putative β-N-acetylhexoaminidases (ID 188060), chitinases (ID: 201136; 136775), and endo-β-N-acetyl-glucosaminidases (ID: 136775, 181728) (Figure S5) were present in D10 up to D15. Their presence corresponded well with the release of N-acetyl glucosamine in chitin digests with these secretomes (Figure S3), and with their observed activity toward *p*NP-N-acetyl-glucosaminide (Figure S7), both under conditions assayed closely mimicking the industrial process conditions. These chitin active enzymes are most likely produced to either allow fungal cell wall modification, attack fungal competitors, or microbial cell wall recycling to recover nitrogen.^48^^,^^49^

### *A. bisporus* secretomes comprise a diverse repertoire of enzymes active on lignin and aromatics

PIII-secretomes and secretomes from axenically cultivated *A. bisporus* were rich in putative enzymes active on lignin and aromatics. This high number of ligninolytic enzymes matched the delignification throughout *A. bisporus* mycelial growth, as it reached 40% (w/w) by the end of the substrate productions and was highest between day 6 and day 13 (Table S2). A substantial number of 28 of such proteins were found, and they are likely involved in the lignin removal previously observed in this lignocellulosic-rich PIII substrate.^12^^,^^16^^,^^50^ Generally, microbial funneling mechanisms, where specifically lignin is first depolymerized and then further metabolized,^51^ heavily rely on extracellular enzymes, in particular on peroxidases and laccases.^52^ Unlike other fungi relying on various types of peroxidases,^51^ only two MnP genes are present in the *A. bisporus* genome.^21^ The protein corresponding to one of these MnPs was found in high abundance in D10, D13 and D15 (MnP, ID 221245). Peroxidases depolymerize lignin via electron transfer^53^ and MnPs having a high redox potential, first oxidize Mn^2+^, which then can be chelated by oxalate and acts as a diffusible charge transfer mediator.^35^ As a consequence, phenoxy- and/or aryl cation radicals are formed, which eventually results in lignin degradation.^35^^,^^52^^,^^54^ Oxalate is known to be a highly produced metabolite of fungi like *A. bisporus*,^55^ and besides its role in chelating ions (e.g., Mn^3+^), it contributes to acidification, and possibly opens up the dense lignocellulose matrix.^56^^,^^57^ Hence, enzymatic recycling of (a surplus of) oxalate by *A. bisporus* can be expected. Already in the early secretomes (D6), one hypothetical *A. bisporus* oxalate decarboxylase (ODC, ID 191066) was detected in high abundance until D15. Based on homology, ODC could also be active as an oxalate oxidase (OXO). Kathiara (2000) characterized this *A. bisporus* protein and showed that it solely targeted oxalate as substrate^58^ to form CO_2_ and formate, pointing at a typical ODC. OXO, on the other hand, converts oxalate to CO_2_ and will generate hydrogen peroxide.^57^

**Figure 3:**
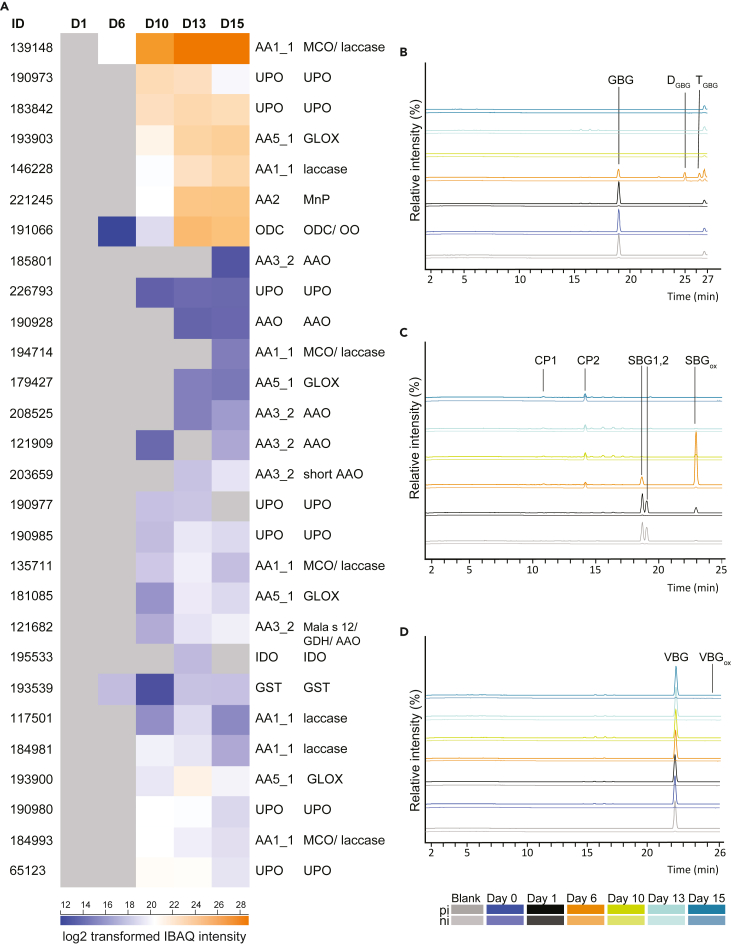
Secretomic profiling of incubations of lignin dimers (A–D) Protein IDs and log2 transformed IBAQ intensities in D6, D10, D13 and D15 active on lignin and aromatics. CAZyme family and subfamily, protein ID and putative functions are provided if available (Joint Genome Institute identifier ‘jgi|Agabi_varbisH97_2’ and Billeti et al. (2012)). MCO = multicopper oxidase; UPO = unspecific peroxidase; ODC = oxalate decarboxylase; MnP = manganese peroxidase; GLX = glyoxal oxidase; IDO = intradiol dioxygenase; AAO = aryl alcohol oxidase. Secretomes were incubated with dimeric lignin models, GBG (B), SBG (C), and VBG (D), and were analyzed by RP-UPLC-MS, corresponding product profiles are shown above in positive (pi) and negative (ni) ionization mode and 16 h of incubation. For abbreviation see Figure 5.

*A. bisporus* proteins from the AA1 family were abundant already in D6 (ID 139148), and (most) were increasingly abundant in D10, D13 and D15 (IDs 221245, 146228, 184981, 117501, 13571; Figure 3). Four of these AA1s were laccases *sensu stricto* (ID 139148, 194714, 135711, 146228). Laccases modify lignin via hydrogen atom abstraction or via electron transfer,^59^ can directly act on phenolic lignin substructures^60^ and can oxidize non-phenolic lignin substructures via mediators.^32^ As a result, and depending on the substrate structures and reaction conditions, lignin (re-)polymerization, lignin-interunit linkage cleavage or Cα-oxidation may occur.^32^^,^^60^^,^^61^ It is still under debate how laccases are involved in fungal delignification,^30^ and whether natural mediators are involved. Still, the observed high abundance of laccases in D10-D15 secretomes, simultaneous with the 40% lignin removal observed for corresponding substrate samples (Table S1), underpins that laccases present in these secretomes are highly relevant for delignification of the plant biomass.

In the early stage of PIII (D6) only one other lignin/aromatic modification-related protein was detected, which was a putative glutathione-S-transferase (GST, ID 193539). This protein was also found in D10 until D15, and since for this *A. bisporus* GST no signal peptide was predicted, this enzyme-protein might have ended up in the secretomes because of fungal cell lysis or through non-classical secretion. Within the GST family, recently one intracellular fungal β-etherase of the white-rot fungus *Dichomitus squalens* has been characterized and shown to cleave the β-*O*-4 ether linkages in lignin via glutathione addition and subsequent reductive cleavage of the ether bond.^62^ However, because the sequence similarity between this *D. squalens* β-etherase and the *A. bisporus* GST is low, the function of the latter GST remains unpredicted. Another putative intracellular protein, only detected in D13, was intradiol dioxygenase (IDO; ID 195533). This *A. bisporus* IDO was somewhat similar based on sequence homology to upregulated dioxygenases found in studies investigating the metabolism of lignin and aromatic compounds in white-rot fungi.^6^^,^^25^^,^^44^ The white-rot dioxygenases have been suggested to catalyze ring cleavage of aromatic compounds as part of their catabolism of aromatic compounds.^6^^,^^25^^,^^44^

Less-known enzymes, in terms of delignification^21^^,^^39^ are the unspecific peroxygenases (UPOs). UPOs have been shown to carry out many reactions, such as epoxidation, alkane hydroxylation, oxidation, halogenation, dealkylation and ether cleavage and combine monooxygenase and peroxidase activities.^35^^,^^38^^,^^63^ Up to now, cleavage of dimeric non-phenolic lignin model compounds has been reported for one UPO.^38^ Kinne suggested that first the UPO demethylates the non-phenolic lignin dimer and that the generated phenoxy radicals are then autocatalytically cleaved. However, in this model system 70% of the products polymerized.^38^ In addition to the observed lignin dimer-cleavage, the demethylation activity of UPOs might allow laccases an easier entry point in lignin depolymerization by generating phenolic lignin structures. The *A. bisporus* genome possesses a surprisingly high number of UPO encoding genes (24), especially compared with ten other white-rot fungi that are known to be efficient lignin degraders, which possess only 3–16 UPO encoding genes.^39^ In secretomes D10-D15, we found seven of these *A. bisporus* UPOs in D10 until D15, and in secretomes from the axenically lab-cultivated *A. bisporus* we also found seven (CDF) or six (CWS) UPOs (Figure 3). The most abundant UPO proteins were the ones with IDs 190973, 183842 and 190980, 65123. It is interesting to note that UPOs 190973 and 183842 were present in higher abundances in D10 than the single MnP observed. At the same time point, substrate delignification was highest (Table S1) making it tempting to speculate that these highly abundant *A. bisporus* UPOs are involved in lignin degradation.

Both peroxidases and UPOs require hydrogen peroxide as co-substrate, which can be delivered by various other enzyme systems. FAD-dependent aryl alcohol oxidases (AAOs; AA3_2) are known to release hydrogen peroxide during oxidation of aromatic alcohols, such as veratryl alcohol, to aldehydes.^39^ Indeed, in our secretomes we found four putative AAOs (IDs: 185801, 190928, 208525, 121909), a short AAO (ID 203659) and another protein which might be an AAO based on structural comparison to known AAOs (ID 121682). Only the latter protein (ID 121682) was found in D10, whereas the others were present only in D13 and/or D15. Hence, to ensure hydrogen peroxide production in the earlier stages of PIII, other enzymes might also have played a role. For example, AA5_1 proteins, such as glyoxal oxidases (GLOX), also produce hydrogen peroxide.^64^^,^^65^^,^^66^ Indeed, in an early stage (D10), four putative *A. bisporus* GLOX proteins were found, with one GLOX (ID 1939030) having a similar abundance as MnP. These proteins were also detected in D13 and D15. Recently it has been shown that laccases can also produce hydrogen peroxide when acting on lignin,^42^ and as discussed above laccases are the most prevalent proteins in the *A. bisporus* secretomes studied, also in secretomes from early stages (Figure 3).

### *A. bisporus* secretomes targeting dimeric lignin model compounds

**Figure 4:**
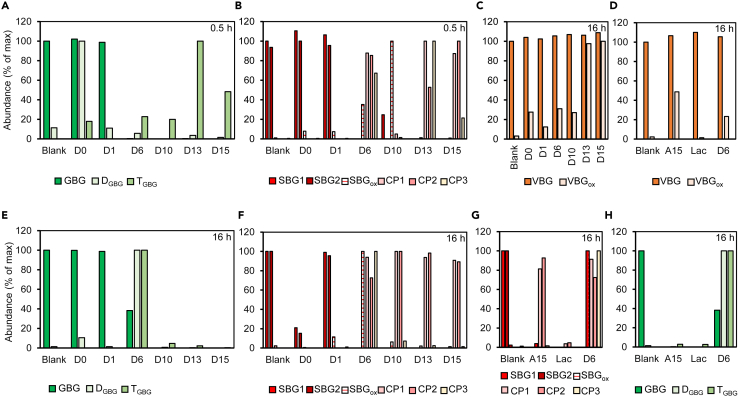
Normalized MS peak areas of reaction products and initial substrates of GBG (A – 0.5 h of incubation, E– 16 h of incubation), SBG (B – 0.5 h of incubation, F – 16 h of incubation), and VBG (C – 16 h of incubation) with *A. bisporus* secretomes obtained from PIII and the axenic secretomes (D – VBG, G - SBG and H-GBG). Areas were obtained from extracted ion chromatograms; for abbreviations and *m*/*z* values see Table 1. For each compound the incubation with the largest peak area was set to 100% = max abundance, and the other abundances were expressed relative to the maximum peak area. In graph D, G and H secretome incubation of day 6 (16 h) was added as comparison to axenic *A. bisporus* secretome (CDF) and commercial laccase.

**Figure 5:**
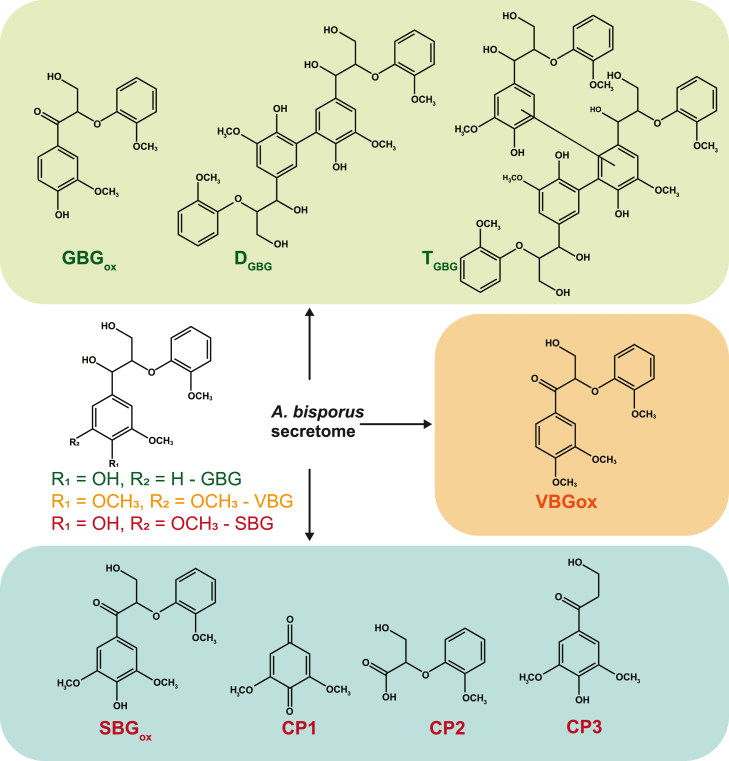
Molecular structures of the used lignin model compounds are shown in the center of the figure (GBG in green, VBG in orange and SBG in red) In the various boxes, the detected reaction products of GBG, VBG and SBG after incubation with *A. bisporus* secretomes are shown. In the green box the GBG reaction products are shown: GBG oxidized (GBGox) and repolymerized structures (D_GBG_, T_GBG_) are indicated. The positions of linkages in GBG dimer (D_GBG_) are based on literature^60^ and of the trimer not indicated (T_GBG_). In the orange box, the sole reaction product of VBG is indicated: the oxidized VBG (VBGox). In the blue box, the SBG reaction products are shown: oxidized SBG (SBGox), the two cleavage products (CP1, CP2), which were annotated based on literature,^38^ and the tentatively annotated cleavage product CP3.

The dimeric lignin model compounds GBG, SBG and VBG were incubated with all secretomes and analyzed by RP-UPLC-MS (Figure 3). Commercial fungal laccase from *Trametes versicolor* was used for comparison, because product profiles of lignin model compounds incubated with laccase were already characterized in our group (Figures 4D, 4G, and 4H).^60^^,^^61^ A summary of substrates and main reaction products after incubation of model dimers with secretomes are shown in Table 1 and Figure 5. Furthermore, semi-quantitative comparison between incubations of reaction products was carried out (Figure 4).

**Table 1:** Most abundant compounds detected by UPLC-PDA-ESI-MS of lignin model structures (SBG, GBG and VBG) and after incubation with *A. bisporus* secretomes

RT (min)	Annotation	Chemical formula	Ion	Observed/theoretical mass	Mass error (ppm)	MS^2^ fragments	λmax (nm)
**GBG**							

18.90	GBG	C_17_H_20_O_6_	[M+Na]^+^	320.12608/320.12599	0.26	295; 329; 296; 325;	279
25.02	D_GBG_	C_34_H_38_O_12_	[M−H]^-^	638.23714/638.23633	2.99	589; 483; 513; 435; 329;	278
26.33	D_GBG_	C_34_H_38_O_12_	[M−H]^-^	638.23708/638.23633	2.90	589; 465;	274
26.74	T_GBG_	C_51_H_56_O_18_	[M−H]^-^	956.34725/956.34667	0.05	ND	ND

VBG							

22.26	VBG	C_18_H_22_O_6_	[M+Na]^+^	334.14140/334.14164	−0.67	309; 343; 310; 299;	278
25.71	VBGox	C_18_H_20_O_6_	[M + H]^+^	332.12595/332.12599	−0.11	315; 303; 285; 291;	ND

SBG							

10.88	CP1	C_8_H_8_O_4_	[M + H]^+^	168.04233/168.04226	0.08	140; 108; 153; 125;	290
14.16	CP2	C_10_H_12_O_5_	[M−H ]^-^	212.06780/212.06848	1.99	193; 195; 196;	274
18.67	CP3	C_11_H_14_O_5_	[M + NH_4_]^+^	226.08398/226.08413	−0.58		274
18.67	SBG	C_18_H_22_O_7_	[M+Na]^+^	350.13636/350.13656	−0.52	325; 359; 230;	274
19.04	SBG	C_18_H_22_O_7_	[M+Na]+	350.13639/350.13656	−0.44	325; 358; 249;	274
22.85	SBGox	C_18_H_20_O_7_	[M+Na]+	348.12070/348.12091	−0.55	353, 341	314

N.D. = not detected. Codes and structures for Annotations are shown in Figure 5.

#### *A. bisporus* secretomes caused polymerization of GBG

The phenolic GBG was not converted upon incubation with D0 and D1 (Figures 3 and 4), which confirmed the absence of lignin-modifying oxidoreductases as concluded from the proteome analysis. In contrast, upon incubations with D10, D13 and D15, >95% and complete conversion of GBG were observed after 0.5 h. In all incubations (0.5h) polymerization products were detected (Figure 4, Table 1). Depending on the secretome and duration of incubation, mainly dimers (D_GBG_) or trimers (T_GBG_) of GBG were observed. Oligomeric structures were nearly absent when GBG was incubated for 16 h with D10, D13, D15 and CDF (Figures 3B and 4E). In these incubations, however, clearly a precipitate was formed (Figure S9) hinting at the formation of larger insoluble GBG oligomers. In accordance with Hilgers (2018),^60^ commercial laccase also generated polymerized GBG complexes after incubation for 16 h (Figure 4). Although indeed (putative) *A. bisporus* laccases were detected in secretomes D6-D15, the observed polymerization of GBG might not be the result of laccase alone, but because of a combination of oxidoreductases. Obviously, (re-)polymerization of lignin-like compounds is not desired in delignification strategies, and not specifically observed to occur as a major process during *A. bisporus* mycelial growth.^12^ Presumably, during fungal growth, other mechanisms, such as immediate take-up of cleavage products by the fungus or other microbes,^6^^,^^67^ might prevent repolymerization.

#### Enzymes in *A. bisporus* secretomes cleaved SBG

The pure phenolic SBG showed two peaks (Rt 18.67 and 19.04) in the LC-MS chromatogram, likely corresponding to two diastereomers (Table 1). SBG was hardly converted by short (0.5 h) incubations with D0 but was after 16 h. Depletion of SBG when incubating with D0 was not expected but could hint at oxidoreductases produced by other microbes present than *A. bisporus*.^47^ Secretomes from D6 until D15 were clearly active toward SBG, resulting in Cα-oxidation (to SBGox) or Cα-aryl cleavage (to CP1 and CP2), or β-*O* cleavage (to CP 3) (Table 1, Figure 5). Annotated cleavage products, CP1 and CP2, were in accordance with Kinne (2011) who incubated SBG with *ae*APO (an UPO)^38^ and mainly observed modifications Cα-oxidation and Cα-aryl cleavage. The highest abundance of SBGox was found in the SBG-D10 digest (0.5 h), whereas for the corresponding longer incubated digest (16 h), SBGox absolutely decreased, and cleavage products CP1 and CP2 were formed. This apparent sequential formation of reaction products might indicate that SBG is first oxidized to SBGox, followed by Cα-aryl cleavage. Furthermore, SBG incubated with D13 and D15, was fully converted within 0.5 h, and clearly showed cleavage products CP1 and CP2 (Figures 4F and 4A). SBG incubated with CDF also showed CP1 and CP2, and to a minor extent SBGox (Figures 5F and 4).

We were able to tentatively identify an SBG cleavage product encoded CP3 (Figures 4 and 5), which based on *m/z* (hydrogen, ammonium and sodium adducts) corresponded to a product formed after β-*O* cleavage of SBG*.* Highest abundances of CP3 was found in SBG-incubations with D13 (0.5h) and D6 (0.5h), and decreased when extending the incubation time, suggesting follow-up reactions to other products. CP3 was also found when incubating SBG with commercial laccase (Figure 4D), and therefore laccase-related proteins in our secretomes might have been involved in generating CP3.

#### The non-phenolic lignin dimer VBG was oxidized by *A. bisporus* secretomes

The non-phenolic lignin dimer VBG was found to be substantially more resistant against conversion by the *A. bisporus* secretomes, as no clear conversion could be observed from the VBG peak intensities. However, when screening for potential VBG reaction products, a Cα-oxidized analogue of VBG (VBGox)^38^^,^^61^ was observed, with a highest relative abundance after 16 h incubation with D13 and D15 (Figures 4 and 5). As expected, no VBGox was found on incubation with commercial laccase, as laccases can only oxidize non-phenolic lignin structures via so-called mediators.^59^ This again suggests that *A. bisporus* also uses other oxidoreductases than laccases to react with lignin and lignin-like compounds. It should be noted that before incubations the secretomes were thoroughly concentrated using 3 kDa filters, possibly removing small mediator-like compounds and/or co-factors/substrate that were originally present in the secretomes.

Overall, the above described reactions clearly indicated that various types of oxidoreductases were present and active toward dimeric lignin model compounds, as has been summarized in Figure 5.

### Conclusions

Secretomes from industrial-scale substrates colonized by *A. bisporus* were quickly and robustly extracted and comprised a diverse mix of lignocellulose-active enzymes. The protein composition of later stage secretomes largely matched secretomes from axenically cultivated *A. bisporus*. A multitude of GHs, CBMs, and oxidoreductases were present in the secretomes, which were involved in plant polysaccharides digestion *in vitro*. Furthermore, a diverse set of enzymes were detected that are known to be involved in lignin and aromatic compound conversion. Temporal dynamic changes of secretomes were observed as multiple laccases, a MnP, and hydrogen peroxide generating enzymes, such as AAOs, and GLOXs, were increasing at later stages when *A. bisporus* fully colonized the substrate. Various UPOs were detected in high abundance and future research is suggested to elucidate their involvement in lignocellulose alteration. Enzymes in secretomes could effectively modify lignin model compounds, as they were either polymerized, oxidized or cleaved. By understanding the *A. bisporus* enzyme machinery we might be able to stir the process to obtain higher substrate-use efficiencies, and learn for future biorefinery applications.

### Limitations of the study

Sampling was carried out at six time-points throughout PIII and incubations with the secretomes were carried out in singlet. During secretome concentration we might have lost low-molecular weight compounds, potentially acting as mediators or cofactors, such as glutathione. Furthermore, we might have missed proteins which were either adsorbed to the substrate or membrane bound. Incubations were only carried out in one buffer, whereas in the real substrate conditions micro-gradients and different environments are likely present and therefore different enzymes might function in the same tunnel-environment. The *in vitro* incubations were done at one pH, as testing many different pH conditions was not feasible regarding the multitude of secretomes, polysaccharides and lignin model compounds tested. Furthermore, hydrogen peroxide could have been added to the incubations to see if peroxidases had higher activities, however we were limited by secretome amount.

## STAR★Methods

### Key resources table

**Table:** 

REAGENT or RESOURCE	SOURCE	IDENTIFIER
**Biological samples**		

Mushroom substrate with *A. bisporus*, PIII	CNC, Milsbeek, The Netherlands	CNC-PIII-072020

**Chemicals, peptides, and recombinant proteins**		

Sodium acetate	Merck/Supelco	CAS 127-09-3
Sodium phosphate	Merck	CAS 13472-35-0
Sodium carbonate	Acros Organics	5668-11-6
Ammonium bicarbonate	Sigma-Aldrich	40867
Ammonium hydroxide solution	Sigma-Aldrich	CAS 1336-21-6
Acetic acid	Sigma-Aldrich	CAS 64-19-7
Acetic anhydride	Sigma-Aldrich	CAS 108-24-7
Acetone	Sigma-Aldrich	CAS 67-64-1
Dichloromethane	Sigma-Aldrich	CAS 75-09-2
Dithiothreitol	TCI	D1071-25g, CAS 3484-12-3
Iodoacetamide	Sigma-Aldrich	CAS 144-48-9
Ammonium acetate	Merck	CAS 631-61-8
UPLC grade water	Biosolve	CAS 7732-18-5
Methanol	Biosolve	CAS 67-56-1
1-methylimidazole	Sigma-Aldrich	CAS 616-47-7
Acetonitrile	Biosolve	CAS 75-05-8
Formic acid	Sigma-Aldrich	CAS 64-18-6
Sulfuric acid	Sigma-Aldrich	CAS 7664-93-9
Sodium borohydride	Sigma-Aldrich	CAS 16940-66-2
1-(4-hydroxy-3-methoxyphenyl)-2-(2-methoxyphenoxy)-1,3-propanediol), (GBG)	TCI chemicals	CAS 7382-59-4, G0233
3-(4-hydroxy-3,5-dimethoxyphenyl)-2-(2-methoxyphenoxy)propane-1,3-diol, (SBG)	ABCR	AB442958, CAS 92409-34-2
1-(3,4-dimethoxyphenyl)-2-(2-methoxyphenoxy)propane-1,3-diol, (VBG)	Alfa Chemistry	ACM10535178, CAS 10535-17-8
4-nitrophenyl-α-D-glucopyranoside	Sigma-Aldrich	N1377-1G, CAS 3767-28-0
4-nitrophenyl-β-D-glucopyranoside	Sigma-Aldrich	N7006-1G, CAS 2492-87-7
4-nitrophenyl acetate	Sigma-Aldrich	N8130-5G, CAS 830-03-5
4-nitrophenyl N-acetyl-β-D-glucosaminide	Sigma-Aldrich	N9376-100mg, CAS 3459-18-5
4-nitrophenyl-β-D-xylopyranoside	Sigma-Aldrich	2132-1G, CAS 2001-96-9
4-nitrophenyl-α-L-arabinopyranoside	Sigma-Aldrich	N3512-1G, CAS 1223-07-0
4-nitrophenyl-α-D-mannopyranoside	Koch-light	CAS 10357-27-4
4-nitrophenyl-β-D-glucuronide	Sigma-Aldrich	CAS 10344-94-2
4-nitrophenol	Sigma-Aldrich	CAS 100-02-7

**Critical commercial assays**		

BCA Protein Assay Kit	Thermo Fisher Scientific	23227

**Experimental models: Organisms/strains**		

*Agaricus bisporus*, strain A15	Plant Breeding, Wageningen University, the Netherlands	http://www.sylvaninc.com/fresh-white-a15/

**Software and algorithms**		

Excel	Microsoft	N/A
Xcalibur 4.1	Thermo Fisher Scientific	https://www.thermofisher.com/order/catalog/product/OPTON-30965
Chromeleon 7.3	Thermo Fisher Scientific	https://www.thermofisher.com/order/catalog/product/CHROMELEON7
ChemDraw 20.0	PerkinElmer Informatics	https://perkinelmerinformatics.com/products/research/chemdraw/
Adobe Illustrator 2022	Adobe	https://www.adobe.com/products/illustrator.html
Perseus_1.6.2.1	Tyanova et al. (2016)^68^	http://coxdocs.org/doku.php?id=perseus:start
MaxQuant 1.6.17	Cox and Mann (2008)^69^	http://coxdocs.org/doku.php?id=maxquant:start
InteractiVenn	Heberle et al. (2015)^70^	http://www.interactivenn.net/

**Other**		

Carboxymethyl-cellulose sodium salt (CMC)	Sigma-Aldrich	C-5678, CAS 9004-32-4
Wheat arabinoxylan (WAX)	Megazyme	P-WAXYM
^13^C lignin isolate from ^13^C wheat straw	IsoLife BV, Laboratory of Food chemistry, WUR	U-60416, ^13^C-LG
Galactomannan WCF	Megazyme	P-GALML
Beechwood xylan	Carl Roth GmbH + Co. KG	CAS: 9014-63-5
Tamarind seed xyloglucan	Megazyme	P-XYGLN
Chitin from shrimp shells	Sigma-Aldrich	C9752-250mg,
D(+)-Glucose	Sigma-Aldrich	G7528-250G
D(+)-Xylose	Sigma-Aldrich	CAS 58-86-6
L(+)-Arabinose	Sigma-Aldrich	CAS 5328-37-0
D(+)-Mannose	Sigma-Aldrich	CAS 3458-28-4
N-Acetyl-D-glucosamine	Sigma-Aldrich	CAS 7512-17-6
Galactose	Sigma-Aldrich	CAS 59-23-4
1,4-β-D-Xylooligosaccharides	Megazyme	O-XBI, O-XTR, O-XTE, O-XPE, O-XPE, O-XHE
Cello-oligosaccharides	Megazyme	C8071
Pullulans	Fluka	p-800-2, p-200-2, p-50-2, p-10-2, p-5-2, p-1-2,
Membrane filter (0.2 μm)	Whatmann GmbH	10410314
Membrane filter (0.45 μm)	Whatmann GmbH	10401614
Diafiltration tubes, IVSS vivaspin 20, 3000 kDa	Sartorius	VSA005
EcoCup – pyrolysis cup	Frontier Lab	PY1-EC80F
Malt extract agar (MEA)	Oxoid	CM0059
Mini-PROTEAN TGX Gels	Bio-Rad Laboratories, Hempel, Hempstead, UK	CAT#4569033
Precision Plus ProteinTM Standard	Bio-Rad Laboratories, Hempel, Hempstead, UK	161 0373
InstantBlueTM	Expedeon, Heidelberg, Germany	ISB1L

### Resource availability

#### Lead contact

Further information and request for resources and reagents should be directed to and will be fulfilled by Mirjam Kabel (mirjam.kabel@wur.nl).

#### Materials availability

Information of materials, and their possible availability, can be requested from the lead contact.

### Method details

#### Sampling of PIII substrate

A concrete PIII-tunnel was filled with wheat straw-based PII-end compost mixed with *A. bisporus* spawned rye kernels at CNC Grondstoffen (Milsbeek, The Netherlands). Five net-bags were filled with the tunnel material (per bag 8 kg ± 10%), and, subsequently, burrowed within the compost in the PIII tunnel. Before mycelial growth and during mycelial growth, after 1, 6, 10, 13, and 15 days, one net bag was collected and thoroughly mixed. All mixed, fresh substrate samples were subjected to secretome extraction.

#### Cultivation of axenically cultivated *A. bisporus*

Freeze dried substrate from PIII day 0 was mixed to milliQ (7.5% DM loading) and boiled for 1.5h under continuous stirring. A part of the substrate suspension was autoclaved as such, and another part, which resembles a more humic substance-, dark-film rich medium, was autoclaved after the remaining wheat straw was sieved out. After complete cool-down of autoclaved media, 500 mL in 1 L Fernbach flasks were axenically inoculated with blended mycelium of pre-cultured (on MEA) *A. bisporus*, A15 (5 mL). The Fernbach flasks were statically incubated for 14 days at 25°C. Negative control of uninoculated substrate medium showed no mycelial growth and mycelium grew in both inoculated substrate media.

#### Extracting *A. bisporus* secretomes from substrate and lab-grown *A. bisporus*

Extracellular enzymes were extracted from mixed, fresh PII end compost, and from PIII compost from D0, D1, D6, D10, D13, D15 by suspending 20 g in 100 mL acetic acid extraction buffer (100 mM; at pH 5.5) and stirring (1 h at 4°C). They represented the crude secretomes extracts from PIII substrates and were coded according to the time point/day when they were collected with: D0, D1, D6, D10, D13, D15. Supernatants of axenically cultivated *A. bisporus* were also subjected to the same purification, washing and concentration as PIII secretomes. The secretome obtained from the substrate medium containing still large wheat-straw particles was coded with CWS and the sieved, dark film and substance-rich medium was coded CDF. PIII substrate supernatants and supernatants from CDF and CWS, which were obtained after decanting the generated mycelium through a sieve, were collected after centrifugation (30000 x g, 30 min, 4°C; Avanti J-26, Beckman Coulter, Indianapolis, IN, USA). Next, the supernatants were filtered through a 0.45 mm filter, followed by a 0.2 mm filter, to remove microbial cells. Further, 20 mL of each filtered liquid was concentrated by 3 kDa centrifugal filters at 4000 x g and 4°C (Heraues Megafuge 16R, Thermo Fisher Scientific, Rockford, IL, USA) for 180 to 210 min, until the filtrate was concentrated 10-fold and washed with extraction buffer. These secretomes were snap-frozen in liquid nitrogen and subsequently stored at −18°C.

#### Protein determination with bicinchoninic acid (BCA)

Protein content in secretomes was estimated using the BCA Protein Assay Kit. Bovine serum albumin was used as a calibration standard in the range of 20–2000 μg/mL.

#### Sodium dodecyl sulfate polyacrylamide gel electrophoresis (SDS-PAGE)

Aliquots of the concentrated secretomes (D0, D1, D6, D10, D13, D15, CDF, and CWS) were heated for 15 min at 95°C in SDS-PAGE sample buffer containing 0.1 M dithiothreitol. Secretome samples were loaded on 12% polyacrylamide gels, together with a low molecular weight protein marker. The gels were subjected to electrophoresis and stained with InstantBlue.

#### Activity of glycosyl hydrolases pNP

Exo-activity of secretomes was tested spectrophotometrically with release of 4-nitrophenol (*p*NP) of *p*NP-labelled substrates. *p*NP-labelled substrates (0.01% (w/v) in acetic acid buffer (100 mM; at pH 5.5) were incubated with diluted secretomes (each mL of secretome contained an equivalent of 0.5 g of the initial substrate). The *p*NP-assay was performed in 96-well plates according to a modified protocol from Jurak (2015)^46^ by adding 40 μL diluted secretome, 10 μL *p*NP-labelled substrate and 50 μL 50 mM sodium acetate buffer (pH 5.0). A calibration curve was generated with 4-nitrophenol (ranging from 5 to 50 nmol). The well-plates were incubated (600 rpm, 1 h, 30°C) and the reaction was stopped by adding 100 μL sodium carbonate (0.25 M). The absorbance of the *p*-nitrophenolate anion was measured at 405 nm with a Tecan infinite F500 microtiter plate reader (Tecan, Männedorf, Switzerland).

#### Plant polysaccharide incubation with secretomes

A diverse set of plant polysaccharides (wheat arabinoxylan, beechwood xylan, carboxymethyl-cellulose (CMC), galactomannan, xyloglucan, chitin from shrimp shell) were solubilized (3 mg/mL) in acetic acid buffer (50 mM, pH 5.5) and incubated with secretomes (D0, D1, D6, D10, D13, D15). The protein loading per incubation was adjusted in such a way that in each Eppendorf the equal amount of protein as in 0.3 g of initial substrate was present. The incubations were carried out at 35°C for 24 h at 45-degree angle. The incubations were stopped by heating to 99°C for 15 min. Control incubations were carried out either without substrate (secretome blanks) or without secretomes (substrate blanks). The reaction mixtures were centrifuged at 15000 × *g* for 15 min, and the clear supernatants were collected and analyzed undiluted by high performance size exclusion chromatography (HPSEC-RI) and diluted (1:1 dilution) with high-performance anion exchange chromatography (HPAEC-PAD).

#### Incubations of secretomes with dimeric lignin model compounds

GBG, VBG and SBG were dissolved (0.05 mM) in sodium phosphate buffer (50 mM, pH 6) by short heating and sonication. Substrates were incubated with secretomes (D0, D1, D6, D10, D13, D15, CDF) and the protein loading per incubation was adjusted in such a way that in each Eppendorf the equal amount of protein as in 0.6 g of initial substrate was present. VBG, GBG and SBG were incubated for 16 h and timepoint samples were taken from GBG and SBG after 0.5 h. The incubations were stopped by adding methanol to a final concentration of 20%. The samples were centrifuged (5000 x g, 5 min, 20°C) prior to analysis and the reaction products were separated using an RP-UHPLC-PDA-MS Thermo Vanquish UHPLC (Thermo Scientific, San Jose, CA, USA) equipped with a pump, degasser, autosampler, coupled to a PDA detector and either a Thermo LTQ VelosPro ion-trap mass spectrometer or a Thermo Q Exactive Focus hybrid quadrupole-Orbitrap mass spectrometer. Samples (1 μL) were injected into an Acquity UPLC BEH C18 column (150 × 2.1 mm, particle size 1.7 μm) with a VanGuard column (5 × 2.1 mm) of the same material (Waters, Milford, MA, USA). The flow rate was 400 μL min^−1^ and the column temperature was 45°C. Water (A) and acetonitrile (B) were used as eluents, and both contained 1% formic acid. The gradient for compound elution was: 0–4 min at 1% (isocratic), 4–25 min from 1 to 32% (linear gradient), 25–26 min from 32 to 100% (linear gradient), 26–30 min at 100% (isocratic), 30–31 min from 100 to 1% (linear gradient), 31–35 min at 1% (isocratic). Full MS data were recorded in both negative and positive ionization mode from *m*/*z* 120–1200. Semi-quantification was based on selective ion extraction based on *m/z* of ions in Table 1.

#### Screening hydrolysis capability of secretomes on polysaccharides HPSEC

High performance size-exclusion chromatography with refractive index detection (HPSEC-RI) was used to determine the molecular weight distribution before and after treating wheat arabinoxylan, xylan, CMC, galactomannan, and xyloglucan with secretomes. The system used was an Utimate 3000 system (Dionex Corp., Sunnyvale, CA, USA)) coupled to a Shodex RI-101 detector (Showa Denko K.K. Tokyo, Japan). Three columns, TSK-Gel Super columns (4000AW, 3000AW, and 2500 AW; 6 ID × 150 mm per column, 6 μm), and a TSK Super AW guard column (6 ID × 40 mm) (Tosoh Bioscience Tokyo, Japan) were used at 55°C. Incubated polysaccharide samples were injected (10 μL) and eluted with 0.2 M sodium nitrate at a flow rate of 0.6 mL min^−1^. Pullulans with molecular weights of 1080, 6100, 9600, 47100, 194000 and 708000 Da were used as standards. The system was controlled with Chromeleon software and data analysis was carried out with Chromeleon software.

#### Screening hydrolysis capability of secretomes on polysaccharides HPAEC

High performance anion exchange chromatography was carried out with Dionex ICS 5000 (Dionex Corp., Sunnyvale, CA, USA) with CarboPac™ PA1 IC column (250 × 2 mm i.d.) and a guard column (50 × 2 mm i.d.). Eluent A was 0.1M sodium hydroxide (NaOH) and eluent B was 1M sodium acetate in 0.1M NaOH and the elution profile was: 0–45 min from 100% A to 38% B; 45–50 min from 38% B to 100% B, 50–55 min 100% B, 55–70 min 100% A, at a flow rate of 0.3 mL/min. The elution of compounds was detected with PAD detector. Control of the system and data analysis was carried out using Chromeleon software. All standards were used at a concentration of 10 μg/mL. Standards of monosugars were glucose, xylose, arabinose, N-acetyl-glucosamine, galactose, mannose, of oligomers were xylotriose, xylotetraose, xylopentaose, xylohexaose, cellobiose, cellotriose, cellotetraose, cellopentaose and cellohexaose.

#### Proteomics sample preparation and analysis

##### In-gel digestion of secretomes

Secretome samples were loaded to SDS-PAGE gels at protein concentrations of 50 μg, 100 μg and 200 μg (determined by BCA) per lane. Samples loaded with 200 μg showed to have the appropriate intensities of protein bands and were used for further proteomics data analysis. Gels were subjected to electrophoresis for 5 min, to remove small humic substance-like compounds. The bands were cut out and sliced in approximately 1 mm^3^ gel pieces. The gel pieces were washed with 0.1 M ammonium bicarbonate:acetonitrile (1:1 v/v) equal to gel volume and this procedure was repeated two times. The washing was discarded, and the gel pieces were shortly dried with SpeedVac, SPD1010 (Thermo Fisher Scientific). The proteins in the gel were then reduced by swelling the gels in 50 μL 0.01M dithiothreitol in 0.1M ammonium bicarbonate for 45 min at 56°C. Then 50 μL of acetonitrile was added, incubated for 5 min and the supernatant was removed. The alkylation was done by adding 50 μL of 0.02 M iodoacetamide in 0.1 M ammonium bicarbonate and incubation for 30 min in the dark. The buffer was removed by washing the gel pieces with 0.1 M ammonium bicarbonate: acetonitrile (1:1 v/v) and the wash was removed, acetonitrile was added and incubated for 5 min, shrinking the gel pieces. The wash was discarded, and the gel pieces were air-dried for 10 min. The proteins were digested by rehydrating them in digestion buffer with sequencing grade porcine trypsin (12.5 ng/μL trypsin in 0.1 M ammonium bicarbonate) by letting them swell for 15 min on ice, the supernatant was removed and then incubated overnight at 37°C. Peptides resulting from the in-gel digestion were extracted from the gel slices by three successive washes using 50mM ammonium bicarbonate, 0.1% formic acid and 50% acetonitrile, respectively. The pooled extracts were dried with SpeedVac and dissolved in 20 μL 0.1% formic acid 2% acetonitrile solvent.

##### Proteomics – Chromatography and mass spectrometry

The extracted digested peptides were injected and separated with a Waters M-class UPLC system (Waters, Milford, USA) on-line connected to a Qexactive^PLUS^ mass spectrometer (ThermoScientific, Palo Alto, USA). Peptides were first collected on a trap column (20 × 15 mm; PepSepC18 Trap, PepSep, Denmark) and subsequently separated on an analytical C18 column (100 mm × 75 μm, PepSep, Denmark) with a 45-min gradient of 6%–30% acetonitrile in 0.1% formic acid (FA) followed by a column clean-up step (isocratic 80% acetonitrile in 0.1% FA), and an equilibration step (isocratic 2% acetonitrile in 0.1% FA), all at a flow rate of 200 nL min^−1^ The eluted peptides were electrosprayed into the mass spectrometer using a Flex-Ion nanoESI Source at +2.4kV. MS and MSMS spectra were collected in top-10 DDA mode selecting charge 2, 3 or 4 ions within *m/z* range of 400–1500 for MS spectra and auto-range for MSMS spectra.

##### Proteomics data processing with MaxQuant

MSMS raw data were processed using MaxQuant software version 1.6.17.0. Settings were mostly default, with variable modifications Oxidation (M) and Acetyl (Protein N-term) and fixed modification carbamidomethyl (C). Data were matched to the *A. bisporus*.

H97.v2 filtered models proteome sequences from JGI containing 10438 entries.^21^

##### Proteomics analysis with Perseus

Hierarchical clustering to generate heatmaps was done with Perseus. Selection of proteins was manually curated and was based on their substrate specificities (Table S1 – proteomics table). A log2 transformation was carried out on IBAQ and hierarchical clustering with Euclidean distance was performed.

#### Substrate compositional analysis

The mixed fresh compost samples were measured for pH and dry weight as previously described.^12^ Lyophilized samples were milled (<1 mm) using an MM 2000 mill (Retsch, Haan, Germany), and analyzed for carbohydrates and lignin. An aliquot of 2 g of milled material was further milled at 600 rpm with a planetary ball mill, PM 100 (Retsch, Haan, Germany), in a zirconium dioxide (ZrO2) jar containing 17 ZrO2 beads (φ 10 mm) for 30 min net milling and after each 10 min milling a 5 min break was held to prevent overheating. The planetary ball milled samples were used for lignin analysis with py-GC-MS.

#### Sugar content and composition

Neutral anhydrocarbohydrate content and composition was determined in duplicate according to Englyst and Cummings (1984).^71^

#### Quantitative lignin analysis by pyrolysis-GC-MS

Lignin content and structural analysis was performed as described by van Erven et al. (2019a),^72^ without modifications for substrate material. The lignin contents were calculated by excluding *p*-hydroxyphenyl units (H- units), since the PIII sample matrix is rich in aromatic amino acids, that upon pyrolysis lead to similar products as lignin-derived *p*-hydroxyphenyl units and *p*-coumarate.

## Data Availability

• All data reported in this paper will be shared by the lead contact upon reasonable request. • This study did not generate original code. • Any additional information required to reanalyze the data reported in this work is available from the lead contact upon reasonable request.

## Supplemental information

Document S1. Figures S1–S9, Tables S2, and S3

Table S1. List of all enzymes in secretomes, related to proteomics data (STAR method) and to Figures 2 and 3
