# Supplementary material for: The secretome of Agaricus bisporus: Temporal dynamics of plant polysaccharides and lignin degradation
Source: iScience. 2023 Jun 9;26(7):107087. doi: 10.1016/j.isci.2023.107087 (PMC10329178; doi:10.1016/j.isci.2023.107087)
Supplement: Document S1. Figures S1–S9, Tables S2, and S3 [file mmc1.pdf]

iScience, Volume *26*

Supplemental information

The secretome of *Agaricus bisporus*: Temporal dynamics of plant polysaccharides and lignin degradation

Katharina Duran, Joris Magnin, Antoine H.P. America, Mao Peng, Roelant Hilgers, Ronald P. de Vries, Johan J.P. Baars, Willem J.H. van Berkel, Thomas W. Kuyper, and Mirjam A. Kabel

Supplemental information

Table S 2 Substrate components (dry weights in tons), and pH, during *A. bisporus* mycelial growth based on the measured weights of the permeable bags, which were retrieved from the tunnel on day 1, day 6, day 10, day 13 and day 15, related to substrate compositional analysis (STAR method). The tunnel contained 100 tons of substrate (wet weight) in the beginning of PIII. Triplicate analysis of dry matter content of bags with duplicate content analysis of lignin and carbohydrates were determined and relative standard deviations ranged between 1.1 to 9.0 %.

	Day 1	Day 6	Day 10	Day 13	Day 15
Cellulose (dry weight in tons)	5.25	4.77	4.38	3.44	4.17
Arabinoxylan (dry weight in tons)	3.13	3.29	2.77	2.56	2.58
Lignin[a] (dry weight in tons)	4.38	4.67	3.56	2.81	2.7
Ash (dry weights in tons)	9.20	8.88	9.13	9.53	8.88
N (dry weights in tons)	0.68	0.67	0.64	0.68	0.65
pH	7.3	7.2	6.7	6.5	6.2

[a] lignin content was determined by quantitative ^{13}C-IS py-GC-MS and H units were excluded

Table S 3 molecular weights of detected proteins with SDS-PAGE, related to sodium dodecyl sulfate polyacrylamide gel electrophoresis (SDS-PAGE) (STAR method).

	Lane	Band No.	Mol. Wt. (kDa)
**PIII_D10**	5.0	1.0	61.8
**PIII_D13**	6.0	1.0	62.6
	6.0	2.0	36.0
	6.0	3.0	25.9
	6.0	4.0	17.5
**PIII_D15**	7.0	1.0	62.7
	7.0	2.0	36.2
	7.0	3.0	26.1
	7.0	4.0	17.6
**Ab_PII**	8.0	1.0	70.1
**Ab_DFPII**	10.0	1.0	74.6

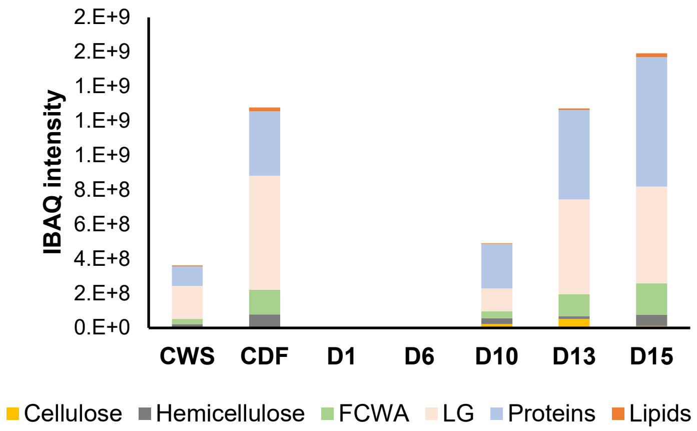

Figure S 1 Sum of IBAQ intensities of putatively annotated enzymes active on cellulose, hemicellulose, fungal/microbial cell walls (FCWA), lignin, protein and lipids in secretomes from PIII and axenic lab-cultivated *A. bisporus* (CDF, CWS), related to Figures 2 and 3.

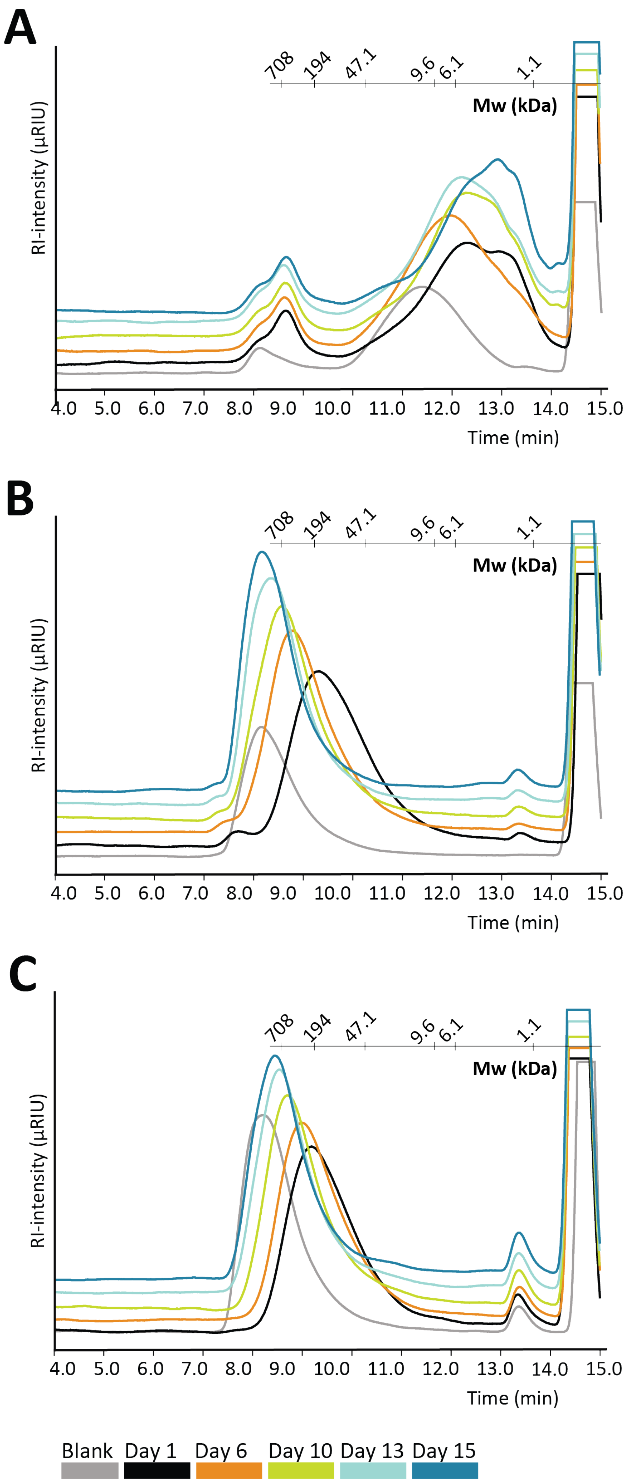

Figure S 2 HPSEC chromatograms of birchwood xylan (A), galactomannan (B) and xyloglucan (C) incubated with secretomes from industrial scale substrate production for *A. bisporus,* related to screening hydrolysis capability on polysaccharides HPSEC (STAR method) and to Figure 2.

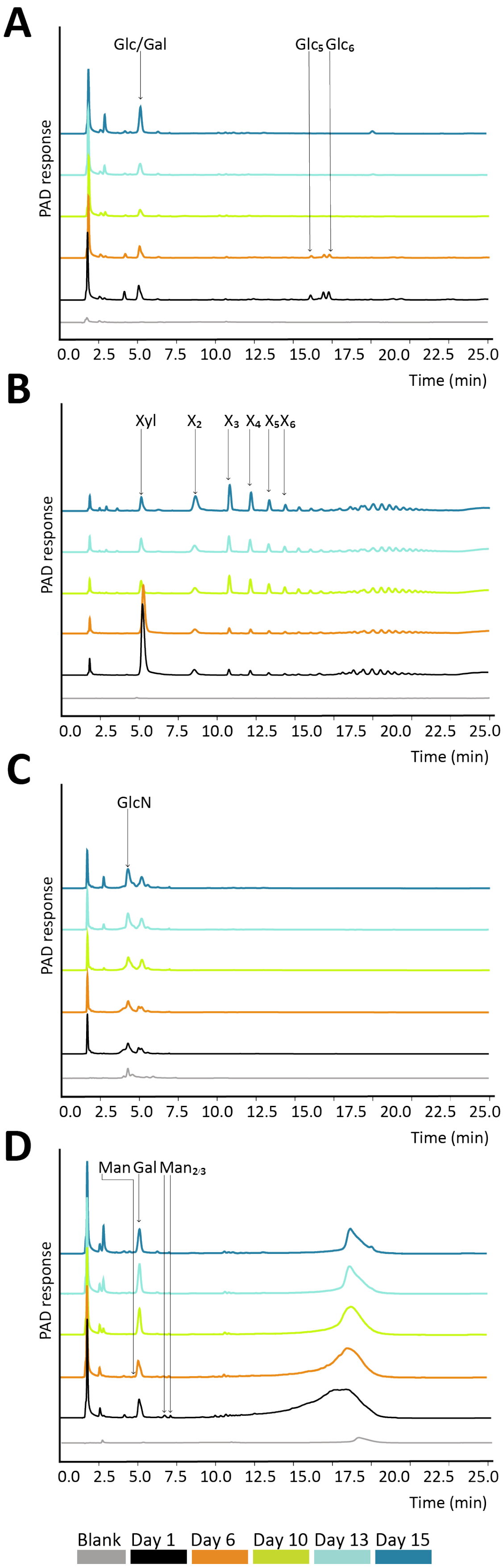

Figure S 3 HPAEC chromatograms of xyloglucan (A), xylan (B) chitin (C), galactomannan (D) incubated with secretomes from industrial scale substrate production for *A. bisporus,* related to screening hydrolysis capability of secretomes on polysaccharides HPAEC (STAR method) and to Figure 3.

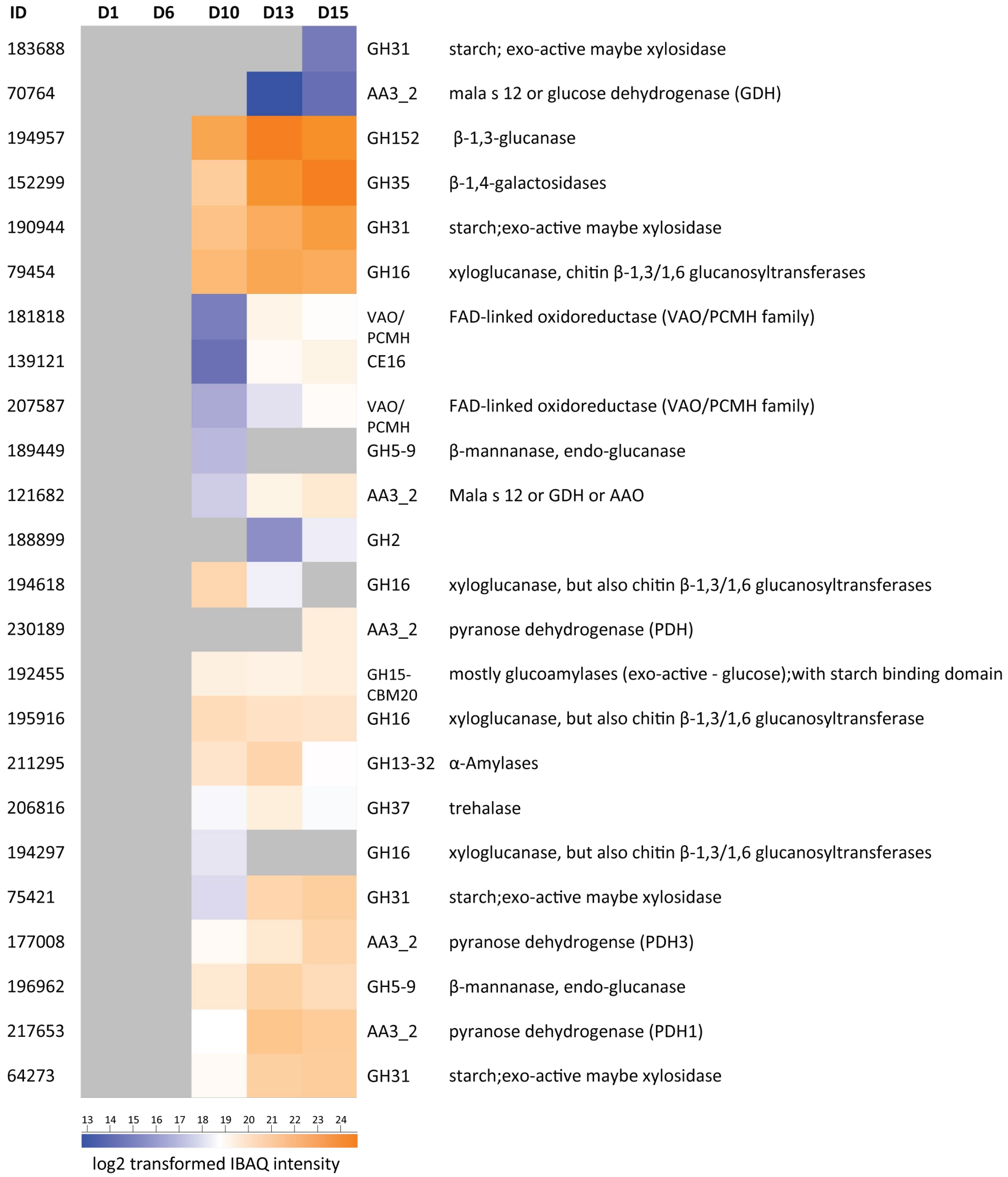

Figure S 4 Secretome profiles of carbohydrates active enzymes, related to Figure 2. Protein IDs and log2 transformed IBAQ intensities in D6, D10, D13 and D15 related to other carbohydrate degradation. Results for other CAZymes are presented in figure S4. CAZymes family and subfamily, protein ID and putative functions are provided if available and based on Joint Genome Institute identifier'jgi|Agabi_varbisH97_2'and Billeti et al.[1]. GH = glycoside hydrolase; CBM = carbohydrate-binding module; AA = auxiliary activities; VAO = vanillyl alcohol oxidase; PCMH = *p*-cresolmethyl hydroxylase.

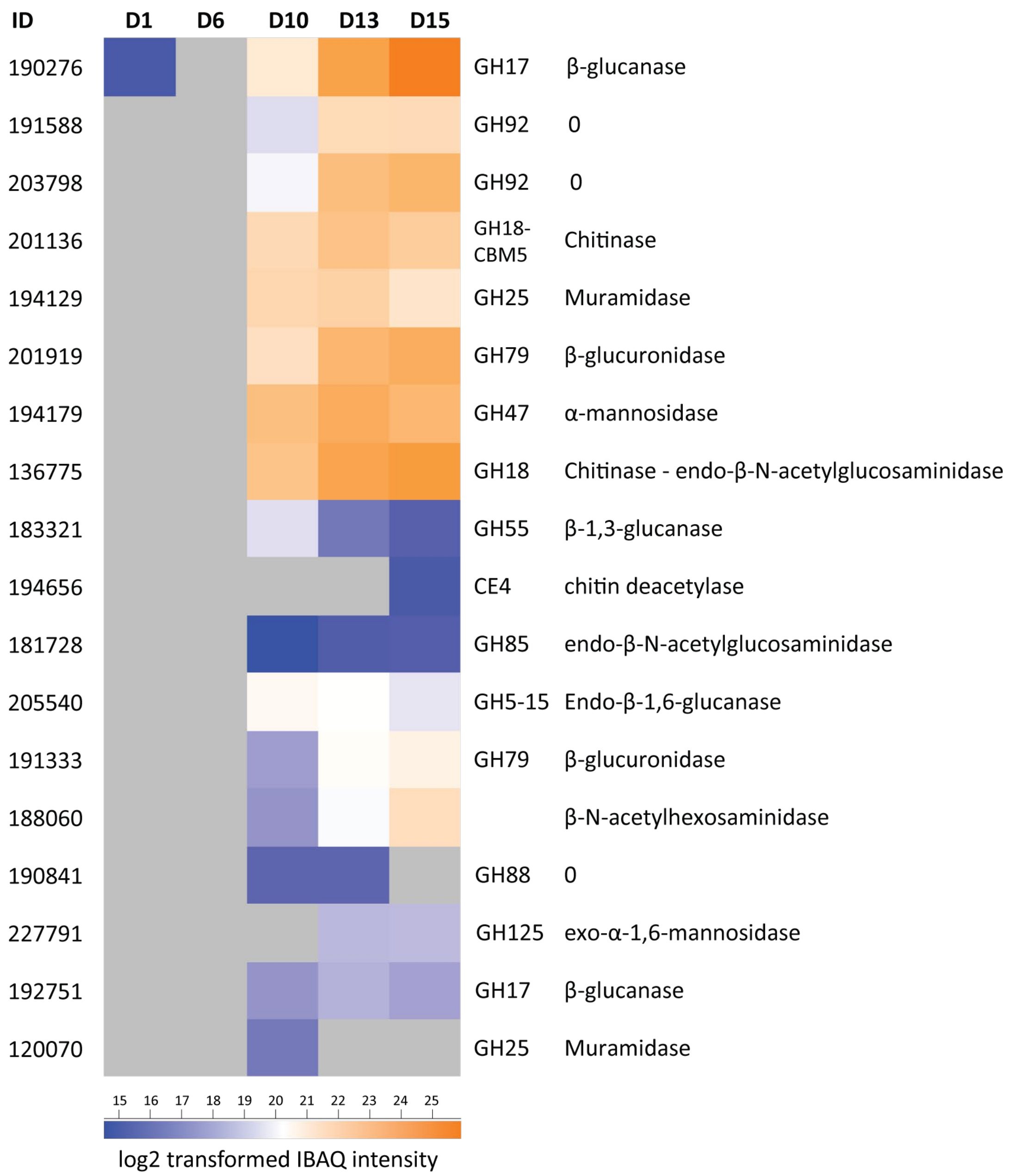

Figure S 5 Secretome profiles of enzymes active on fungal and microbial cell walls, related to Figure 2 and 3. Protein IDs and log2 transformed IBAQ intensities in D6, D10, D13 and D15 related to fungal- and microbial- cell wall degradation. Results for other CAZymes are presented in figure S4. CAZymes family and subfamily, protein ID and putative functions are provided if available and based on Joint Genome Institute identifier'jgi|Agabi_varbisH97_2'and Billeti et al.[1]. GH = glycoside hydrolase; CBM = carbohydrate-binding module.

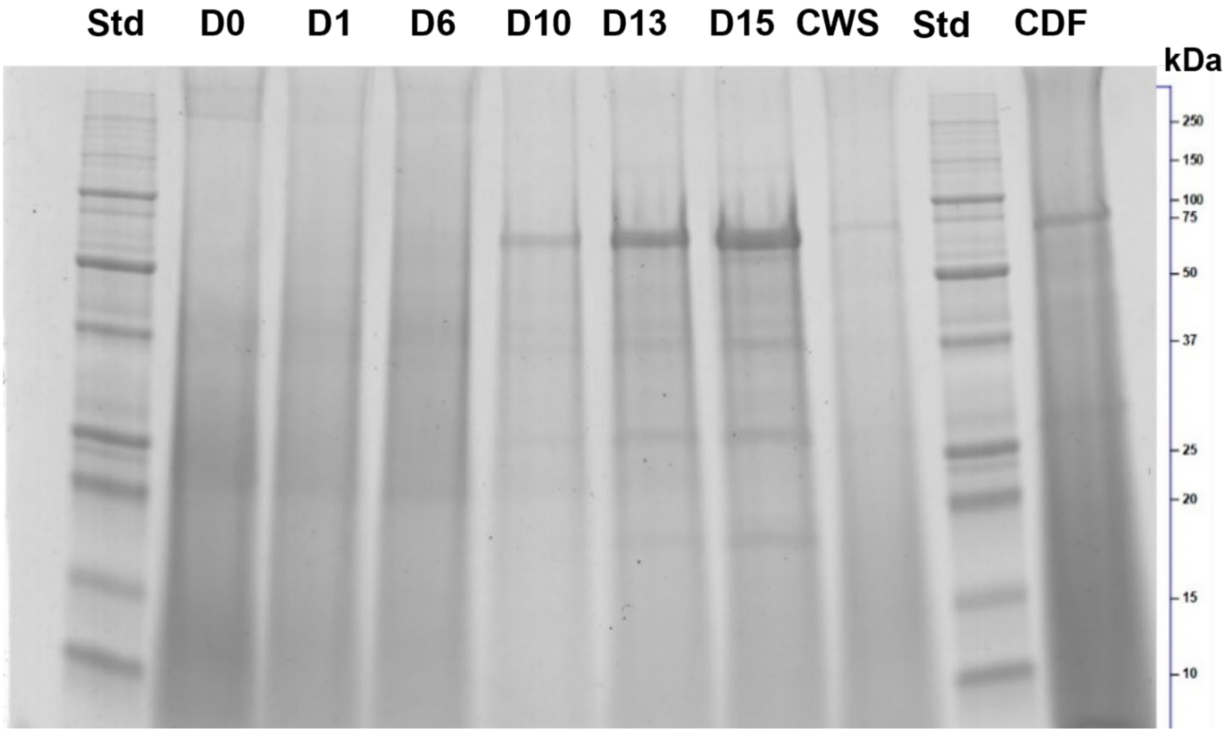

Figure S 6 SDS-PAGE analysis of secretomes, related to sodium dodecyl sulfate polyacrylamide gel electrophoresis (SDS-PAGE) (STAR method). The protein markers are indicated with Std.

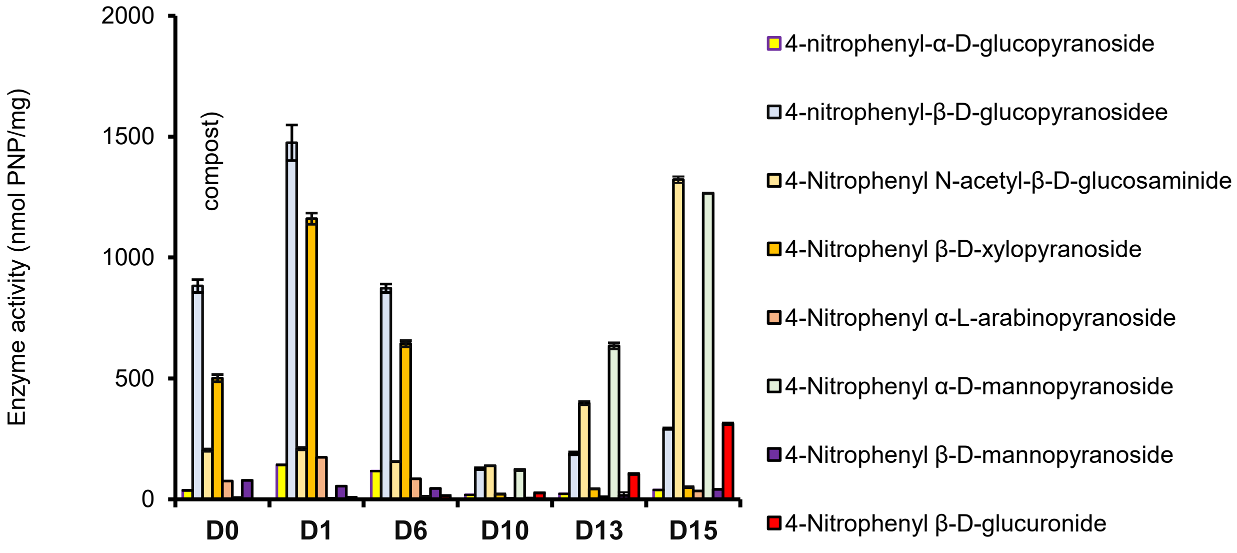

Figure S 7 Enzyme activities on *p*NP labelled substrates by PIII secretomes, related to activity of glycosyl hydrolases pNP (STAR method) and related to Figure 2.

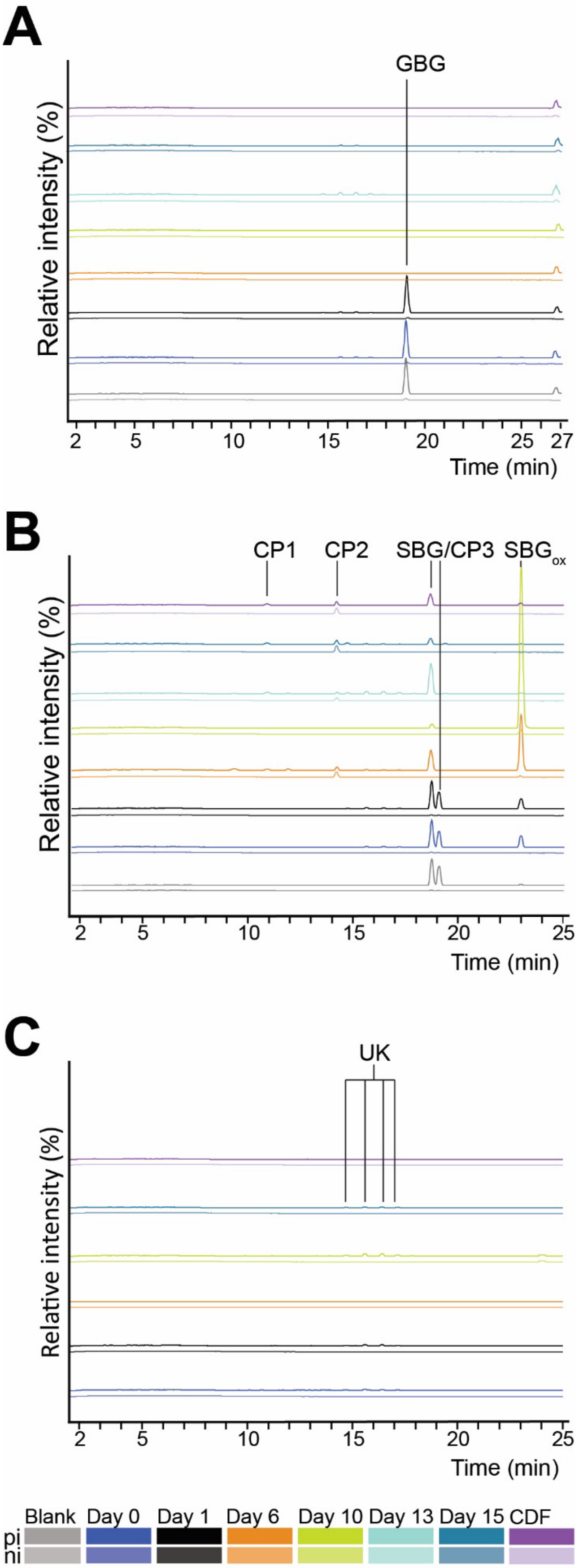

Figure S 8 UPLC-MS chromatograms, related to incubations of secretomes with dimeric lignin model compounds (STAR methods) and Figure 3. Secretomes were incubated with dimeric lignin models, GBG (**B**), SBG (**C**), and VBG (**D**), and were analyzed by RP-UPLC-MS, corresponding product profiles are shown above in positive (pi) and negative (ni) ionization mode and 0.5 h of incubation. For abbreviation see figure 5.

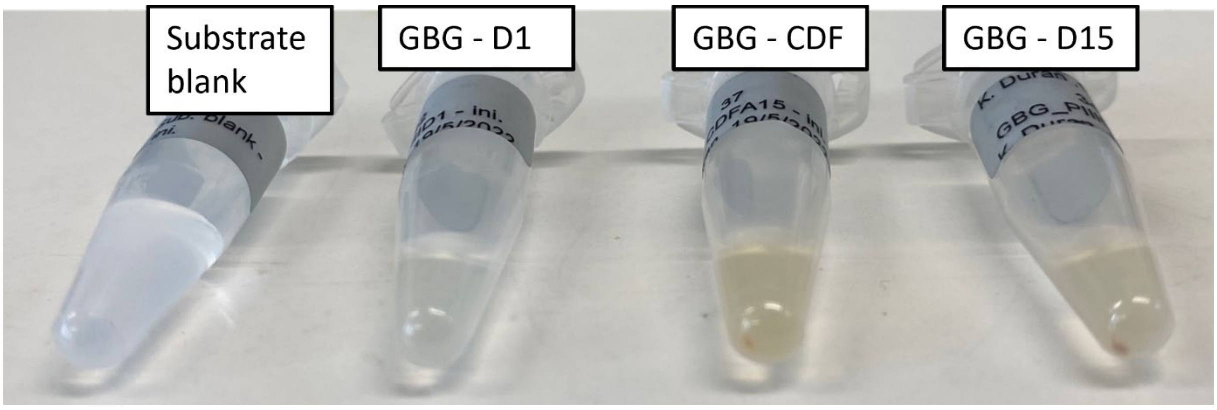

Figure S 9 Picture of lignin model incubations, related to Figure 3. GBG substrate blanks and GBG incubations with D1, DCF and D15. D1 did not cause insoluble pellet and CDF and D15 caused pellet formation.

References Supplementary

1. Billette, C., Murat, C., Kerrigan, R.W., de Vries, R.P., Grigoriev, I. V., Burton, K.S., Subramanian, V., Lapidus, A., Lombard, V., Baker, A.R., et al. (2012). Genome sequence of the button mushroom *Agaricus bisporus* reveals mechanisms governing adaptation to a humic-rich ecological niche. Proc. Natl. Acad. Sci *109*, 17501–17506. 10.1073/pnas.1206847109.
